# Inhibition of heat-induced apoptosis in rat small intestine and IEC-6 cells through the AKT signaling pathway

**DOI:** 10.1186/1746-6148-9-241

**Published:** 2013-12-02

**Authors:** Zhimin Gao, Fenghua Liu, Peng Yin, Changrong Wan, Shasha He, Xiaoxi Liu, Hong Zhao, Tao Liu, Jianqin Xu, Shining Guo

**Affiliations:** 1 College of Veterinary Medicine, South China Agricultural University, Tianhe, Guangzhou, Guangdong 510642, R. P China; 2 Department of Animal Science and Technology, Beijing University of Agriculture, Beijing 102206, P. R China; 3 College of Veterinary Medicine, China Agricultural University, No. 2, West Yuanmingyuan Road, Beijing 100193, P. R China

**Keywords:** Heat stress, Apoptosis, AKT, Small intestine, IEC-6 cells, Rat

## Abstract

**Background:**

As the world warms up, heat stress is becoming a major cause of economic loss in the livestock industry. Long-time exposure of animals to hyperthermia causes extensive cell apoptosis, which is harmful to them. AKT and AKT-related serine–threonine kinases are known to be involved in signaling cascades that regulate cell survival, but the mechanism remains elusive. In the present study, we demonstrate that phosphoinositide 3-kinase (PI3K) /AKT signal pathway provides protection against apoptosis induced by heat stress to ascertain the key point for treatment.

**Results:**

Under heat stress, rats showed increased shedding of intestinal epithelial cells. These rats also had elevated levels of serum cortisol and improved expression of heat shock proteins (Hsp27, Hsp70 and Hsp90) in response to heat stress. Apoptosis analysis by TUNEL assay revealed a higher number of villi epithelial cells that were undergoing apoptosis in heat-treated rats than in the normal control. This is supported by gene expression analysis, which showed an increased ratio of Bax/Bcl-2 (p < 0.05), an important indicator of apoptosis. During heat-induced apoptosis, more AKTs were activated, showing increased phosphorylation. An increase of BAD phosphorylation, which is an inhibitory modification, ensued. In rat IEC-6 cell line, a significant higher level of AKT phosphorylation was observed at 2 h after heat exposure. This coincided with a marked reduction of apoptosis.

**Conclusion:**

Together, these results suggest that heat stress caused damages to rat jejunum and induced apoptosis to a greater degree. HSPs and pro-survival factors were involved in response to heat stress. Among them, AKT played a key role in inhibiting heat-induced apoptosis.

## Background

Heat stress is a common stressful factor that affects many biological systems. Research over the past decade has demonstrated that hyperthermia causes various damages to the animal body, including injuries in the central nervous system [1] and adrenal glands [2], reduction of thyroid physiology in lactating cows [3], and gastrointestinal hyperpermeability [4]. The integrity (both structural and functional) of the small intestine is essential for absorption of nutrients. However, it can also be jeopardized by hyperthermia. Especially, hyperthermia causes damages to the tips of intestinal villi, where epithelial cells renewal requires a large amount of energy [5]. Under high temperature, the blood flow to the small intestine is reduced significantly to increase that to essential organs such as brain and cardiac. This greatly impairs the small intestinal villus epithelial cells [5,6], and induces excessive apoptosis of them.

Apoptosis, also known as programmed cell death, is a physiological suicide mechanism by which cells die under strict control [7,8]. It is characterized by specific features, including nuclear fragmentation, DNA fragmentation, and apoptotic body formation. The formed apoptotic bodies are rapidly phagocytosed by neighboring cells or macrophages, without causing a damaging inflammatory response [9,10]. A lot of researches demonstrate that as a critical media of apoptosis, heat stress would induce apoptosis in cells [11,12]. Although apoptosis is a normal physiological process, in excess it is pathologic [13].

PI3K/AKT signaling has been reported to block apoptosis induced by diverse apoptotic stimuli, and promotes cell survival in a variety of apoptotic paradigms [14-16]. However, little is known about its role in heat-induced apoptosis. In this signaling pathway, AKT is the primary mediator. It has a number of downstream substrates that may contribute to tumor genesis. In the presence of survival factors, AKT becomes activated, which in turn phosphorylates and inactivates components of the apoptotic machinery, such as Bad. Bad and other Bcl-2 family members are known to function as critical regulators of apoptosis pathways, acting to either inhibit (Bcl-2, Bcl-xl) or promote (Bak, Bad) cell death [17]. Thus, AKT may serve to repress apoptosis by inhibiting the activities of pro-apoptotic proteins.

From our previous study on heat-stress, we hypothesized that cell apoptosis in small intestine play crucial role under state of heat-tress. To investigate heat-induced apoptosis in rat small intestine and IEC-6 cells, and to examine the role of AKT in this apoptosis, the rats were simulated in hyperthermia. After heat exposure, the morphological changes were detected by electron microscopes. Apoptotic cells were examined by TUNEL assay. Our results suggest an effect of AKT on suppressing apoptosis triggered by heat stress, so that AKT would be as a target for treatment for a more general aim of this study is to improve animal growth.

## Method

### Animals

All experimental protocols were approved by the Committee for the Care and Use of Experimental Animals at Beijing University of Agriculture.

Twelve healthy male Sprague Dawley (SD) rats (BW200 ± 20 g) were obtained from Beijing Vital River Laboratory, Animal Technology Co., Beijing, China, and raised (25°C, 60% relative humidity) for 7 days freedom to water and food. Then the rats were divided into two groups, control and heat stress group.

### Treatment and sampling

Rats in the control group were raised at an atmosphere of 25°C, 60% relative humidity (RH); the heat stress group was housed at 40°C, 60% RH between 11:00 am and 1:00 pm daily for three consecutive days. Rats from both groups were sacrificed by broken neck immediately after the heat exposure period.

Rat body temperatures were recorded after heat exposure using a thermistor probe connected to a digital thermometer. Their body weights were also recorded. Blood samples were immediately collected after execution, and centrifuged at 3,000 × g for 10 min. Intestinal tissue samples of duodenum, jejunum and ileum were collected afterwards. The samples of each tissue were divided into two parts: One was fixed in 10% buffered formalin phosphate for histological analysis; the other was stored at -80°C.

### IEC-6 cell culture, cell treatment and morphology observation

IEC-6 cells (CRL21592, obtained from Peking Union Medical College) were grown in Dulbecco’s Modified Eagle Medium (DMEM) containing 5% (v/v) fetal bovine serum (HyClone, USA), 2 mg/L insulin, 50 IU/ml penicillin and 50 mg/ml streptomycin. The cells in control group were strictly regulated at 37°C and 5% CO_2_, while cells in heat-stressed group were exposed to 42°C and 5% CO_2_ for 15 minutes, 30 minutes, 1 hour, 2 hours, 4 hours and 8 hours. Changes in cell morphology were observed using phase-contrast microscope (IX71/IX2, Olympus).

### Morphological analysis and apoptosis detection

The formalin-fixed tissues were embedded in paraffin and transversely sectioned (5 mm thick). After deparaffinization and dehydration, the sections were stained by hematoxylin and eosin.

Apoptotic cells were visualized with TUNEL kit (Promega G7310, Madison, WI, USA) following the manufacturer’s protocol. Briefly, after deparaffinization and dehydration, protein digestion was done by incubating tissue sections in 20 mg/ml proteinase K for 15 minutes at room temperature. Sufficient rTdT reaction mix was prepared before for both control and stress groups. One hundred microliters of reaction mix per slide will adequately cover the section. After the reaction of Terminal Deoxynucleotidyl Transferase Recombinant(rTdT), sections were covered with plastic cover slips, incubating at 37°C for 60 minutes inside a humidified chamber; reactions were terminated by immersing the slides in sodium citrate (SSC). Then, sections were incubating in Horseradish Peroxidase (HPR). Finally, the sections were colored by diaminobenzidine (DAB) at room temperature. Microstructures of the small intestine were observed using a BH2 Olympus microscope (DP71, Olympus, Japan).

### RT-PCR

Expression of HSP70, HSP90 and HSP27were quantitatively determined using real-time PCR. Quantitative PCR analysis was carried out using the DNA Engine Mx3000P® fluorescence detection system against a double- stranded DNA - specific fluorescent dye (Stratagene, USA) according to optimized PCR protocols. β-actin was amplified in parallel with the target genes providing a control.

The system included 1 μl cDNA, 10 μl mix, 0.3 μl Rox, 6.7 μl Diethypyrocarbonate (DEPC) for every sample, then PCR was carried out as follows: one cycle of denaturation at 94°C for 5 min; denaturation at 94°C for 30 s, annealing at 60°C 30 s; a total of 40 cycles for 1 min at 72°C for 30 s; followed by one cycle of 5 min at 72°C for the final extension. The primes were listed in the Table 1.

**Table 1:** Primer sequences for real-time PCR

**Description**	**Accession number**	**Primer sequence**	**Product (bp)**
β-actin	NM_031144	Forward: TTGTCCCTGTATGC CTCTGG	218
		Reverse: ATGTCACGCACGATTTCCC	
HSP27	NM_031970.3	Forward: GGCAAGCACGAAGAAAGG	269
		Reverse: GATGGGTAGCAAGCTGAAGG	
HSP70	NM_031971.2	Forward: CGTGCCCGCCTACTTCA	280
		Reverse: CACCAGCCGGTTGTCGA	
HSP90	NM_175761.2	Forward: GTCCCGGTGCGGTTAGTCACG	70
		Reverse: TTGGGTCTGGGTTTCCTCAGGC	
Bcl-2	NM_001191.2	Forward: CTGGGAGGAGAAGATGC	126
		Reverse: ACCTTTGTTCCACGACCCATAG	
Bax	NM_017059.2	Forward: CAGGACGCATCCACCAAGAA	114
		Reverse: GGGTCCCGAAGTAGGAAAGG	

### Protein extraction and Western blot

Tissus and cells were harvested and lysed in lysis buffer (5 μL phosphatase inhibitors, 1 μL protease inhibitor and 5 μL 100 mM PMSF). After incubation (30 min, on ice), lysates were centrifuged (10,000 g, 5 min, 4°C). The supernatant was removed and the protein concentration was measured using a BCA protein assay reagent according to the manufacturer’s instructions.

Equivalent amounts of protein were subjected to SDS-PAGE electrophoresis and then electroblotted onto nitrocellulose membrane, in which process, the concentration of gel is 12%. The membrane was incubated with primary antibody and then IRDye 800CW-conjugated secondary antibody, and the infrared fluorescence image was obtained using Odyssey infrared imaging system (Li-Cor Bioscience, Lincoln, NE, USA).

The antibody used in the western blot: rabbit anti-AKT (1:1000; #4685 Cell Signaling Technology Inc.), rabbit anti-phospho-AKT (1:2000; #4060 Cell Signaling Technology Inc.), rabbit anti-Bad (1:1000; #9239 Cell Signaling Technology Inc.), rabbit anti-phospho-Bad (1:1000; #4366 Cell Signaling Technology Inc.), GAPDH(1:1000; Cell Signaling Technology Inc.).

### Statistical analysis

All data are presented as the mean ± SD. Statistical analysis was performed by independent-sample T-tests using SPSS 11.5 AP-value of < 0.05 was considered significant. Microarray analysis was conducted using a Molecule Annotation System.

## Result and discussion

### Assessment of heat stress

**Figure 1:**
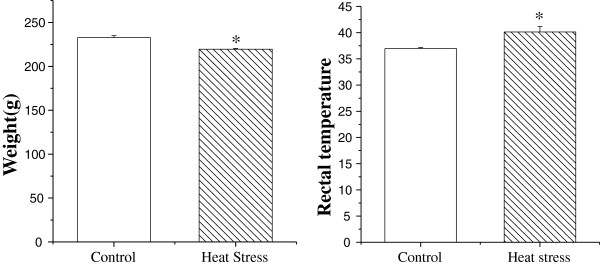
**Heat stress induced serious affect on physiology.** The weight of rats decreased significantly after heat stress, and rectal temperature significantly increased. Values represent the mean±SD, n=6 rats for each group. *p<0.05 control versus heat stress.

In research of heat stress, the rectal temperature is generally considered one of indexes of heat stress assessment, after heat exposure, the rectal temperature increased significantly [18], and our study showed similar result of previous research (Figure 1).

**Figure 2:**
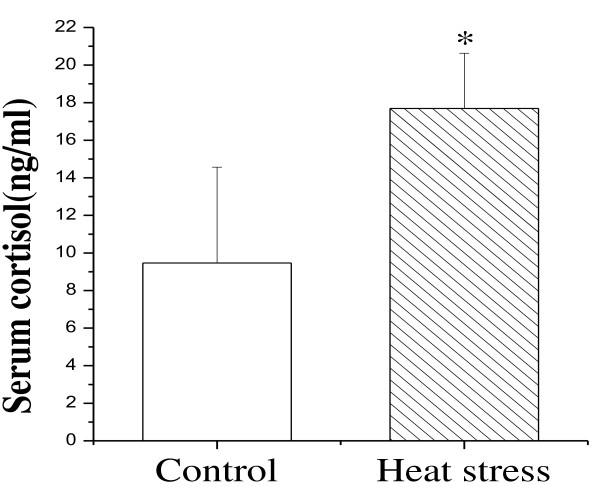
**Changes of glucocorticoid concentrations between control and heat-stressed group.** In the stressed group, the serum corticosterone increased significantly. Values represent the mean ±SD, n=6 rats for each group. *p<0.05 heat-stressed versus control.

**Figure 3:**
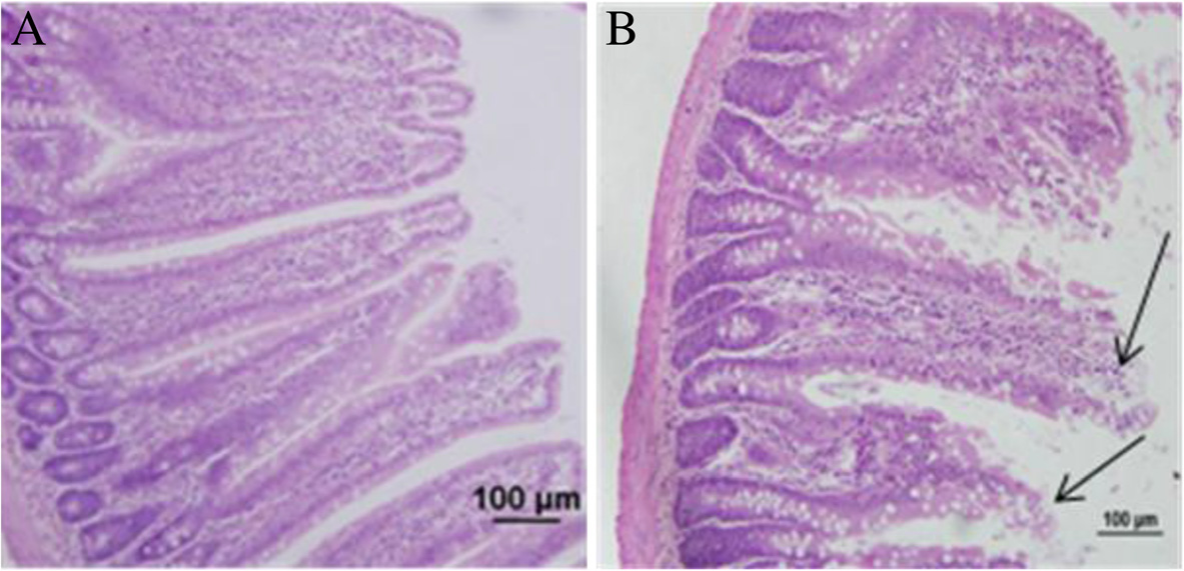
**Photomicrographs of hematoxylin and eosin-stained sections of control (A) and heat-treated (B) stressed groups.** After treated by hyperthermia, the integrity of small intestine was damaged (jejunum), with desquamation at the top of the intestinal villi and exposure of the lamina propria(indicated by arrows).

The intestinal epithelium provides a physical barrier between the luminal contents and the interior environment of the body and protects the body against entrance of bacteria, bacterial toxins, and other unwanted macromolecules. In rats, about 30% of the total blood flow goes to the small intestine. However, when they are exposed to heat for a long time, this rate reduces significantly to increase the cerebral blood flow for heat dissipation [19]. The decrease of blood flow to the small intestine in this situation results in ischemia and shedding of intestinal epithelial cells, and it was most serious on the third day [6]. The damage on intestinal would result in the reduction of food, and then induce decrease of body weight (Figure 1). Another index, glucocorticoid, which is critical for successful adaptation [20], is considered to be a good indicator of stress response intensity, particularly in its acute phase. Thus, the increased glucocorticoid levels observed in heat-treated rats in this study may be indicative of higher stress during heat exposure (Figure 2). Similarly, our results revealed severe shedding of epithelial cells in heat-treated rats, suggesting that the small intestines of these rats were damaged by heat stress (Figure 3).

**Figure 4:**
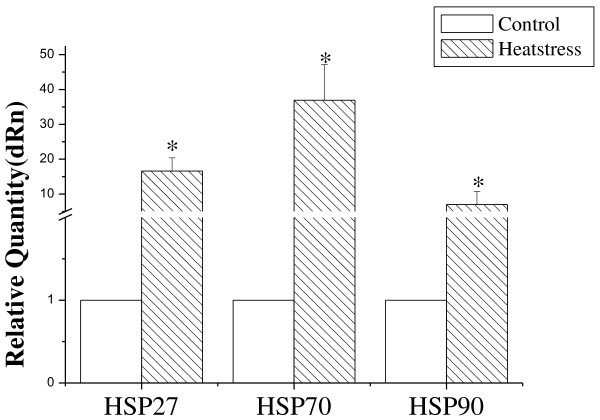
**Expression of rat HSP genes which were detected by RT-PCR.** The expression of HSP genes increased significantly after heat exposure. Values represent the mean ± SD, n = 6 rats for each group. *p <0.05 heat-stressed versus control.

Since first reported by Ritossa [21], knowledge about HSPs has been increasing. They are known to limit the damage caused by stress, and promote cellular recovery [22]. Consistently, in this study, the expression of HSP 27, HSP 70 and HSP 90 were elevated after heat exposure. These data underscore the role of HSPs in cellular resistance to heat and in heat adaption (Figure 4).

### Apoptosis induced by heat stress

**Figure 5:**
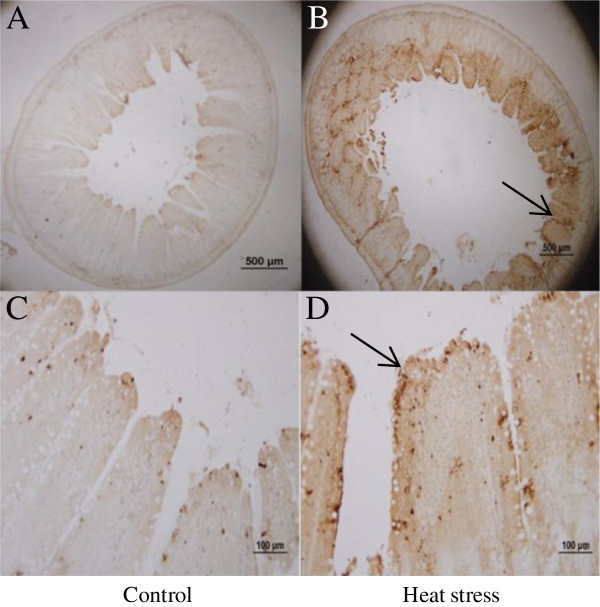
**Apoptosis in jejunum of non-treated and heat-treated rats.** The apoptotic cells of stressed group **(B,D)** was increased after stress compared with control **(A,C)** (indicated by arrows).(**A**,**B** 40×; **C**,**D** 200×).

**Figure 6:**
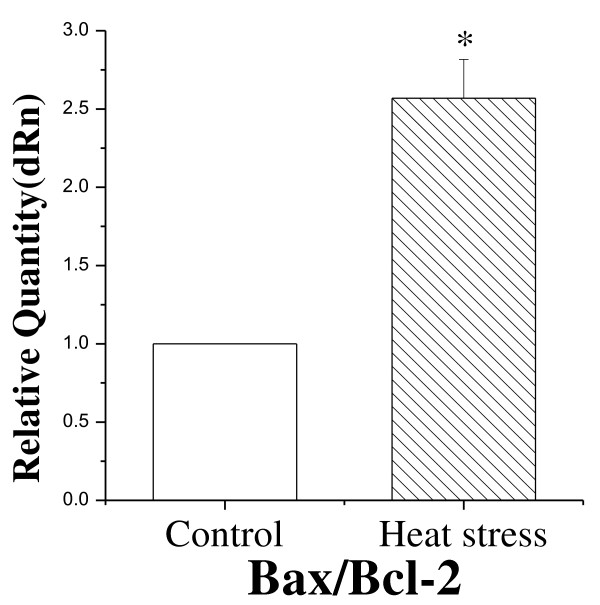
**Ratio of Bax/Bcl-2 before and after heat stress detected by RT-PCR.** The ratio Bax/Bcl-2 was significantly higher in heat-treated group than in control. Values represent the mean ± SD, n = 6 rats for each group. *p <0.05 heat-stressed versus control.

During apoptosis, apoptotic cells finally break up into “apoptotic bodies” and are phagocytosed by phagocytes or neighboring cells. TUNEL assay is always considered a usual method to detect apoptotic cells [23-25]. To proof the effect of heat stress caused on the cells, it is crucial to detect apoptotic cells, the results that revealed much higher apoptotic rates in heat-treated rats was proofed heat stress caused damage to intestine cells (Figure 5). Moreover, apoptotic cells in these rats migrated to the bottom of villi. Given that the intestinal epithelial cells differentiate from cells in the crypts at the bottom of villi, these results may suggest a much more severe apoptosis in heat-treated rats. Gene expression analysis further supported the TUNEL assay results by showing a higher Bax/Bcl-2 ratio in heat-treated rats. Bcl-2 family members are key regulators of apoptosis. They can either repress (eg. Bcl-2) or promote (eg. Bax) apoptosis [26]. However, heterodimerization between Bax and Bcl-2 may negate the function of either protein. The relative ratio of Bax/Bcl-2 is known to be an important indicator of apoptosis. An excess of Bcl-2 homodimers promotes cell survival [27], whereas an excess of Bax homodimers promotes apoptosis. Thus, the ratio of Bax/Bcl-2 determines whether a cell will die or survive [28]. In agreement with a previous report [29], our results showed that the ratio of Bax/Bcl-2 increased significantly after heat exposure. This ratio was obtained on the third day of heat treatment, because it is the time when the most serious damages were observed in our preliminary experiments. Interestingly, in this study, the significant increase of Bax/Bcl-2 ratio in heat-treated rats was coincident with a considerable amount of villi epithelial cells undergoing apoptosis (Figure 6). Therefore, our data are consistent with the observation that a considerably increased Bax/Bcl-2 ratio is associated with the peak period of apoptosis. Taken together, these results suggest that heat stress induced the apoptosis of villi epithelial cells, accompanied by down-regulation of Bcl-2 gene and up-regulation of Bax gene.

### Expression of AKT in vivo and in vitro

**Figure 7:**
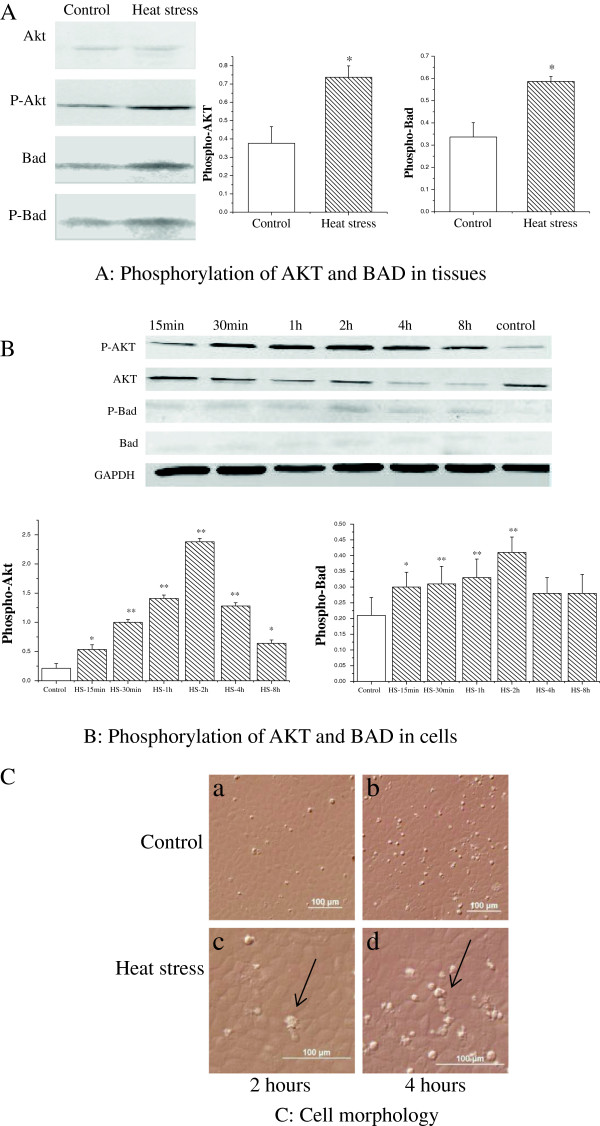
**Effect of AKT on heat-induced apoptosis. (A)** Phosphorylation of AKT and BAD in rat small intestine was significantly increased after heat exposure. **(B)** AKT and Bad proteins extracted from rat IEC-6 cells. Cells were treated with heat for 15 min, 30 min, 1 h, 2 h, 4 h and 8 h, respectively. The phosphorylation of AKT and BAD were significantly higher at 2 h of heat exposure than at other time points. **(C)** was observed after heat exposure. Compared to control, heat stress caused damages to cell morphology. The damage was more serious at 4 h **(b**,**d)** of heat exposure than at 2 h**(a,c)**(**a**,**b** 200×; **c**,**d** 400×). *p <0.05, ** p <0.01 heat-stressed versus control.

AKT plays key roles in regulating cell growth, survival and metabolism [30]. It was first discovered as an oncogene within the mouse leukemia virus [31,32] and as a homolog of protein kinase C [33]. Thereafter, there have been many exciting breakthroughs elucidating the mechanism of upstream regulation of AKT [34-36]. AKT promotes cell survival through the phosphoinositide 3-kinase (PI3K) pathway. After phosphorylation, AKT phosphorylates Bad (serine-136) and inhibits the pro-apoptosis effect [15], inactive Bad promotes apoptosis by binding to Bcl-xl protein, phosphorylated Bad in turn interacts with 14-3-3 proteins to promote cell survival [37]. In the present study, we found a key role for the activation of PI3K/AKT in the apoptosis induced by heat stress. This was accompanied by an increase in Bad phosphorylation, which is an inhibitory modification of Bad (Figure 7). Together, these results indicate that in response to heat stress, AKT is activated to inhibit apoptosis and promote cell survival. Although AKT may play roles in the whole process of heat treatment, our in vitro study revealed significantly higher phosphorylation of AKT and BAD at 2 h of heat exposure (Figure 7). At this time, the numbers of apoptotic cells was also significantly reduced, compared with the number of apoptotic cells after heat stressed 4 hour (Figure 7). The previous research of our lab demonstrated that heat-stress induced apoptosis in IEC-6 cells after treated 4 hours, and lead to necrosis treated more time [38]. The present study suggested that PI3K/AKT pathway protects villi epithelial cells from apoptosis at certain points in the apoptotic process. Collectively, our data support a role of AKT in antagonizing apoptosis of villi epithelial cells through the PI3K/AKT pathway. Heat stress may stimulate the activity of AKT to repress apoptosis.

## Conclusions

In conclusion, the results in this present study suggest that heat stress affected growth of rats, caused damages to the small intestine and induced shipping and apoptosis of epithelial cells. This study also demonstrates that PI3K/AKT signal pathway was involved in the resistance mechanisms of apoptosis induced heat stress. In addition, in IEC-6 cell lines, a significant higher level of AKT phosphorylation was observed at 2 h after heat exposure, this indicates the PI3K/AKT pathway has an effect on an early period.

## Competing interests

The authors declare that they have no competing interests.

## Authors’ contributions

Z was the student who conducted the study, and wrote the manuscript. P, C, S, H, T and X collected the materials and culture cell lines. F, X and S reviewed the manuscript and the quality of the written English. All authors read and approved the final manuscript.

## Authors’ information

Zhimin Gao and Fenghua Liu are Joint first authors.
